# Safety, Tolerability, Pharmacokinetics, and Food Effects on TAC‐302 in Healthy Participants: Randomized, Double‐Blind, Placebo‐Controlled, Single‐Dose and Multiple‐Dose Studies

**DOI:** 10.1002/cpdd.776

**Published:** 2020-01-22

**Authors:** Shogo Sesoko, Jinhong Huang, Takashige Okayama, Erika Nishida, Kazuhisa Miyoshi

**Affiliations:** ^1^ Sosenkai Clinic Edogawa Mizue Edogawa‐ku Tokyo Japan; ^2^ Pharmacovigilance Department Taiho Pharmaceutical Co. Ltd. Uchikanda Chiyoda‐ku Tokyo Japan; ^3^ Pharmacokinetics Research Laboratories Taiho Pharmaceutical Co. Ltd. Okubo Tsukuba Ibaraki Japan; ^4^ Clinical Development II Department Taiho Pharmaceutical Co. Ltd. Uchikanda Chiyoda‐ku Tokyo Japan

**Keywords:** dysuria, food effects, lower urinary tract dysfunction, pharmacokinetics, TAC‐302

## Abstract

TAC‐302 stimulates neurite outgrowth activity and is expected to restore urinary function in patients with lower urinary tract dysfunction. We conducted 2 phase 1, randomized, placebo‐controlled studies to confirm the safety and pharmacokinetics (PK) of TAC‐302 in healthy adult Japanese male volunteers. In the first‐in‐human single‐dose study (n = 60), TAC‐302 was administered at doses from 100 to 1200 mg after an overnight fast. The effects of a meal on the PK of TAC‐302 400 mg were also examined. A multiple‐dose study (n = 36) evaluated the effects of meal fat content on the PK of single doses of TAC‐302 (100, 200, or 400 mg) and multiple doses of TAC‐302 administered for 5 days (100, 200, and 400 mg twice daily). TAC‐302 showed linear PK up to doses of 1200 mg in the fasting state, and across the dose range of 100–400 mg in the fed state. No accumulation of TAC‐302 was observed. Food, particularly with high fat content, increased TAC‐302 plasma concentrations. No differences were observed in the adverse event incidence between the TAC‐302 and placebo groups in either study. TAC‐302 showed a wide safety margin.

The lower urinary tract, comprising the bladder and urethra, has both a storage function (accumulation of urine in the bladder) and a voiding function (excretion of accumulated urine).^1^ Disorders of these functions are collectively referred to as lower urinary tract dysfunction (LUTD), and the symptoms of LUTD are referred to as lower urinary tract symptoms, which are classed as storage, voiding, or postmicturition symptoms.^1^

During recent years, many new treatments have been developed for overactive bladder.^2^ Conversely, underactive bladder—a disease concept that is currently being investigated—has no pertinent therapeutic agents.^3^ Recent clinical data suggest that partial denervation of the bladder and other lower urinary tract tissue, due to various factors, may have an important influence on LUTD progression.^4^ Such denervation may therefore be a key target in the treatment of bladder dysfunction and lower urinary tract symptoms.^4^ Indeed, previous reports showed that some cyclohexenonic long‐chain fatty alcohols have neurotrophic activity on cultured neurons from the cerebral cortex.^5^

TAC‐302 3‐(15‐hydroxypentadecyl)‐2,4,4‐trimethyl‐2‐cyclohexen‐1‐one is a novel and potent cyclohexenonic long‐chain fatty alcohol derivative that stimulates neurite outgrowth activity in cultured neurons.^5^ It has been reported that cyclohexenoic long‐chain fatty alcohol improved bladder function in rats.^6^, ^7^, ^8^ Via such neurotrophic activity, TAC‐302 is expected to counteract partial denervation of the bladder and lower urinary tract tissue and restore urinary function.^9^ Based on the nonclinical data, the estimated main elimination pathway of TAC‐302 is metabolism, and a hydroxyl group of TAC‐302 was metabolized to the carboxylic acid. The estimated main metabolic enzyme responsible for TAC‐302 metabolism in human is alcohol dehydrogenase class III, which is dependent on nicotinamide adenine dinucleotide and the optimum pH of which is in the alkaline condition. Indeed, in in vitro cultured rat dorsal root ganglion neurons, TAC‐302 dose dependently and significantly increased neurite outgrowth.^9^ In an in vivo rat model of bladder outlet obstruction, 3 or 30 mg/kg of TAC‐302 twice daily for 4 weeks significantly (*P* < .05) reduced nonvoiding contractions and residual urine volume. Significant negative correlations were evident between innervation area score and nonvoiding contractions, and between innervation area score and residual urine volume.^9^ Thus, TAC‐302 is expected to emerge as a treatment for LUTD—particularly dysuria—that is markedly different from existing therapeutic agents for patients with refractory overactive bladder or underactive bladder.^9^

As a first‐in‐human study, we conducted a phase 1 single‐dose study to confirm the safety, tolerability, and pharmacokinetics (PK) of TAC‐302 at doses from 100 to 1200 mg, and to determine the effects of food on TAC‐302 PK after administration of a single oral 400‐mg dose in healthy Japanese adult male volunteers. Subsequently, we conducted a phase 1 multiple‐dose study to assess the safety, tolerability, and PK of TAC‐302 400 mg. We also evaluated the effects of food and the effects of meal fat content on TAC‐302 PK.

## Methods

### Study Design

#### Single‐Dose Study

This was a phase 1, single‐center, randomized, double‐blind, placebo‐controlled, crossover comparison study. The study was conducted between January 2013 and May 2013 at Maruyama Hospital, Shizuoka, Japan.

A prestudy evaluation was conducted at each step before the full evaluation. One participant each (a healthy adult male volunteer) was randomly assigned to receive TAC‐302 as a single dose or placebo. After confirming 24‐hour safety in these 2 participants, another 8 participants were enrolled in the TAC‐302 group, and 2 participants were enrolled in the placebo group. The PK data obtained during the prestudy evaluation in those 2 participants were used for the main analysis.

**Figure 1:**
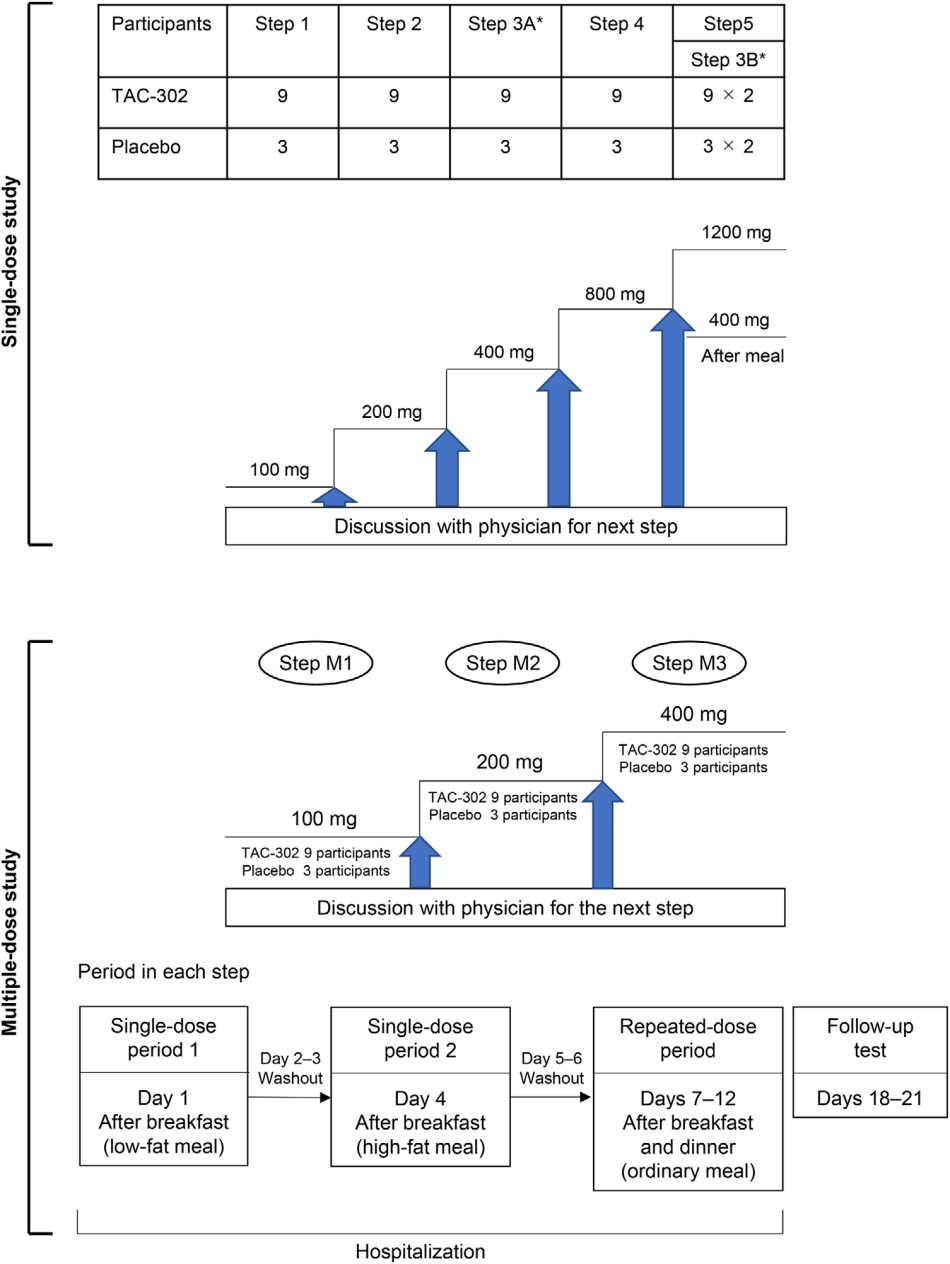
Design of single‐ and multiple‐dose studies. All doses were administered in the fasting state, except for 400 mg, which was administered after an ordinary meal. *Same participants.

PK was evaluated after a single administration of TAC‐302 to 60 healthy adult male volunteers at 5 steps (12 participants in each step: 9 participants in each TAC‐302 group, and 3 in each placebo group): step 1, 100 mg; step 2, 200 mg; steps 3A and 3B, 400 mg; step 4, 800 mg; and step 5, 1200 mg (Figure 1).

Participants were randomized using the placebo group as a control, and the study was conducted under double‐blind conditions. All participants, the sponsor group (ie, project manager and monitors), and site staff (investigators and clinical coordinators) were blind to the treatment, except for the pharmacist who allocated the investigational drugs.

In steps 1, 2, 3A, 4, and 5, TAC‐302 was administered as a single dose (100–1200 mg) after an overnight fast. The effects of food on the PK of TAC‐302 were examined in step 3B, at which a single dose of TAC‐302 400 mg was administered to the same participants as in step 3A. In step 3B, conducted after step 4 for the crossover comparison, TAC‐302 was administered within 30 minutes after an ordinary meal. An ordinary meal was defined as having a total caloric content of ∼700 kcal, of which fat comprised ∼25% (Table 1).

**Table 1:** Meal Content

	Type	Total Calorie Content (kcal)	Proportion of Total Energy as Fat (%)
Single‐dose study	Ordinary meal	∼700	∼25
Multiple‐dose study	Low‐fat meal	≤450	≤20
	High‐fat meal	≥900	≥35
	Ordinary meal	∼700	∼25

In every step, TAC‐302 was administered with a 250‐mL glass of water at around 9:00 am. Additionally, participants were not permitted to eat or drink anything other than the provided study diet and water from 1 hour prior to TAC‐302 administration to 4 hours after study drug administration. Participants were not allowed to lie down for 4 hours after study drug administration unless it was necessary for evaluation.

#### Multiple‐Dose Study

This phase 1, clinical pharmacology study was a single‐center, randomized, double‐blind, parallel‐group, placebo‐controlled trial conducted between October 2013 and May 2014 at Maruyama Hospital. The effects of TAC‐302 were evaluated in 36 healthy adult male volunteers (12 participants at each dose: 9 participants in each TAC‐302 group, and 3 in each placebo group) after single‐dose oral administration of TAC‐302 100, 200, or 400 mg at 30 minutes after a low‐fat meal (day 1, single‐dose period 1) and at 30 minutes after a high‐fat meal (day 4, single‐dose period 2; Figure 1).

The effects of TAC‐302 were also evaluated after twice‐daily multiple‐dose oral administration for 5 days (100 mg bid, step M1; 200 mg bid, step M2; 400 mg bid, step M3) under fed conditions with ordinary meals (days 7‐12; Figure 1). Based on the result of a 4‐week repeated‐dose toxicity study in rats, TAC‐302 400 mg twice daily was set as the maximum dose so that participant safety could be ensured with multiple‐dose administration. The study drug was administered with a 150‐mL glass of water at approximately 9:00 am and 9:00 pm. Participants were not permitted to eat or drink anything other than the provided study diet and water from 1 hour prior to study drug administration up to 4 hours after study drug administration. On days 1, 4, 7, and 11, participants were not allowed to lie down for 4 hours after study drug administration, unless it was necessary for evaluation. The low‐fat meal in single‐dose period 1 and the high‐fat meal in single‐dose period 2 were set with reference to criteria in bioequivalence study guidelines for generic drugs (Table 1).

### Study Participants

All participants in the single‐dose and multiple‐dose studies were healthy adult Japanese male volunteers aged 20 to 40 years.

#### Principal Inclusion Criteria

The principal inclusion criteria in both studies were body weight ≥50 kg and body mass index (BMI) ≥18.5 and <25.0 kg/m^2^ at screening. Additionally, all participants were judged by the investigator or subinvestigator to be healthy, based on physical examination findings (subjective symptoms and objective findings), blood pressure, pulse rate, body temperature, 12‐lead electrocardiogram, and laboratory tests (hematology, clinical chemistry, endocrinology, and urinalysis) at screening and before administration of the study drug.

#### Principal Exclusion Criteria

In both studies, participants with a complication, history of drug hypersensitivity or allergy, or illicit drug use or alcohol dependence; participants who had used drugs (including over‐the‐counter drugs) or nutraceuticals (including supplements) within 7 days before the start of study drug administration; individuals who smoked cigarettes; and participants who did not use contraceptive measures were excluded.

### Study Outcomes

#### Single‐Dose Study

The objectives of the single‐dose study were to examine the PK, safety, and tolerability of single‐dose oral administration of TAC‐302 and determine the effects of food on TAC‐302 PK after administration of a single oral 400‐mg dose.

#### Multiple‐Dose Study

The objectives of the multiple‐dose study were to examine the PK, safety, and tolerability of multiple‐dose oral administration of TAC‐302 and determine, in an exploratory manner, the effects of food (specifically, fat content) on TAC‐302 PK after single‐dose oral administration.

### Study Ethics

All screened individuals provided written informed consent to participate, and the studies were conducted in accordance with the ethical principles of the Declaration of Helsinki and Good Clinical Practice. The locally appointed ethics committee/institutional review board of the participating site, the Maruyama Hospital Institutional Review Board, approved the research protocol.

### Study Assessments and Statistics

#### Sample Size

The sample size for both studies (60 participants and 36 participants in the single‐dose and multiple‐dose studies, respectively) was determined such that safety and PK can be assessed while allowing for variation. For each step in both studies, the PK analysis set comprised only the 9 participants in each TAC‐302 dosage group. In both studies, the safety analysis set comprised all participants who received at least 1 dose of TAC‐302 or placebo. For all safety assessments, adverse event (AE) and adverse drug reaction (ADR) data were collected using system organ class and preferred terms from the *Medical Dictionary for Regulatory Activities*, Version 16.1.

#### Single‐Dose Study—PK

Blood sampling (5 mL per sample) in steps 1, 2, 3A, 3B, 4, and 5 was performed immediately before administration and at 0.5, 1, 1.5, 2, 4, 6, 8, 12, 24, and 48 hours after administration. Urine sampling was performed immediately before administration and at 0 to 6, 6 to 12, 12 to 24, and 24 to 48 hours after administration. TAC‐302 concentrations in plasma and urine were measured by validated bioanalytical methods using liquid chromatography–tandem mass spectrometry (LC‐MS/MS). To establish the lower limit of quantitation (LLOQ), a 50‐µL sample of human plasma was used, and the LLOQ was set at 0.100 ng/mL. The LLOQ for urine samples was 1 ng/mL.

Using standard noncompartmental measures, PK parameters were calculated using plasma and urine concentrations of TAC‐302 on each administration day. The influence of food on TAC‐302 PK was evaluated: maximum plasma concentration (C_max_), time to C_max_ (t_max_), area under the plasma concentration–time curve (AUC) from time 0 to 24 hours (AUC_0‐24h_), AUC from time 0 to 48 hours (AUC_0‐48h_), AUC from time 0 to infinity (AUC_0–inf_), elimination half‐life, and mean residence time from steps 3A and 3B were compared between fasting and fed conditions. The urinary excretion rate was calculated based on the dose, urinary concentration of TAC‐302, and urine volume. Renal clearance was calculated from the urinary concentration of TAC‐302, urine volume, and AUC_0–24h_. Calculation of PK parameters for plasma concentrations was performed using the PK analysis software Phoenix WinNonlin Professional, Version 6.1 (Certara USA, Inc., Princeton, New Jersey).

#### Multiple‐Dose Study—PK

Sampling (2 mL per sample) for those receiving a single dose occurred immediately before administration after breakfast and at 0.5, 1, 1.5, 2, 4, 6, 8, 12, and 24 hours after administration on days 1 and 4. Sampling (2 mL per sample) for those receiving multiple doses took place immediately before administration after breakfast and at 0.5, 1, 1.5, 2, 4, 6, 8, and 12 hours after breakfast on days 7 and 11. Samples were taken only immediately before drug administration, after breakfast, on days 8, 9, and 10. The linearity of TAC‐302 PK after the first dose on day 7 was evaluated using linear regression. The influence of food intake (fat content) on TAC‐302 PK was evaluated by comparing C_max_, t_max_, and AUC_0–12h_ after a low‐fat, high‐fat, and ordinary meal (Table 1).

#### Statistical Analyses

All statistical analyses were conducted using SAS software, Version 9.2 (SAS Institute Inc., Cary, North Carolina). Results were collected in Microsoft Excel 2010 (Microsoft Corporation, Redmond, Washington).

TAC‐302 concentrations in plasma and urine were measured by validated bioanalytical methods (validation items: including within‐ and between‐day variability), and Triple Quad 5500 (AB SCIEX, Redwood City, California) and Shimadzu liquid chromatography system (Shimadzu Corporation, Kyoto, Japan) were used as LC‐MS/MS. TAC‐302 stable isotope (TAC‐302‐d_6_) contained 6 deuterium atoms and was used as the internal standard (IS) for the measurement. Unison UK‐C18 (Imtact, 2.0 mm i.d. × 50 mm, 3 µm) was used as analytical column, and positive monitor ions for TAC‐302 and TAC‐302‐d_6_ were m/z 365 to m/z 111 (Q1 to Q3) and m/z 371 to m/z 114 (Q1 to Q3), respectively; 0.1% formic acid and acetonitrile were used as mobile phase. The lower limits of quantification (LLOQs) in plasma and urine were set at 0.1 ng/mL and 1 ng/mL, respectively.

The plasma samples (50 µL) were mixed with 50 µL of methanol and 100 µL of acetonitrile including IS, and centrifuged at 15 000 g (or 5000 × *g*) for 2 minutes at 5°C. The supernatants were transferred into a filter and filtrated by centrifugation at 15 000 × *g* for 2 minutes at 5°C. The resultant filtrates were injected to LC‐MS/MS. The urine samples (25 µL) were mixed with 225 µL of 50% ethanol and 250 µL of acetonitrile including IS, and centrifuged at 15 000 × *g* for 2 minutes at 5°C. The supernatants were injected to LC‐MS/MS.

For linear model analysis, C_max_ and AUC_0‐12h_ were plotted according to the following hypothesis: *Y* = *aX* + *b* (*X*: dose, *Y*: C_max_ or AUC_0‐12h_). We judged the parameters to be linear when the 95% confidence intervals of *b* included 0 and the lack‐of‐fit test was not significant (significance level, .05).

For PK parameters of TAC‐302 after a low‐fat or high‐fat meal, C_max_ and AUC data were converted to a common logarithm, and a 2‐sided t‐test was carried out. For t_max_, the Wilcoxon signed‐rank test was carried out without converting to a common logarithm. Fat content was considered to have no influence on PK parameters if there were no significant differences (significance level, 5%) in the analyses of those parameters.

## Results

### Participant Disposition and Characteristics

#### Single‐Dose Study

For each study step, 12 participants were randomized. As no participants were ineligible, the number of eligible participants at each step was 9 (75.0%) in the TAC‐302 group and 3 (25.0%) in the placebo group. Overall, in the 45 participants who received TAC‐302, the mean (± standard deviation [SD]) age was 30.3 (5.9) years, body weight was 62.7 (6.7) kg, height was 171.2 (5.3) cm, and BMI was 21.4 (1.8) kg/m^2^. Corresponding values in the 15 participants who received placebo were age, 30.2 (4.4) years; body weight, 63.8 (7.6) kg; height, 172.8 (6.9) cm; and BMI, 21.3 (1.7) kg/m^2^.

#### Multiple‐Dose Study

For each study step, 12 participants were randomized. As no participants were ineligible, the number of eligible participants at each step was 9 (75.0%) in the TAC‐302 group and 3 (25.0%) in the placebo group. Overall, 27 participants received TAC‐302 and 9 received placebo; there were no discontinuations. Participant characteristics are listed in Table 2.

**Table 2:** Participant Characteristics (Multiple‐Dose Study)

	Step Timing of Administration	1 (TAC‐302 100 mg) After Food Intake		2 (TAC‐302 200 mg) After Food Intake		3 (TAC‐302 400 mg) After Food Intake	
	Treatment Group	TAC‐302 Group (N = 9)	Placebo Group (N = 3)	TAC‐302 Group (N = 9)	Placebo Group (N = 3)	TAC‐302 Group (N = 9)	Placebo Group (N = 3)
Sex	Female, n (%)	0 (0.0)	0 (0.0)	0 (0.0)	0 (0.0)	0 (0.0)	0 (0.0)
	Male, n (%)	9 (100.0)	3 (100.0)	9 (100.0)	3 (100.0)	9 (100.0)	3 (100.0)
Age, y	Mean (SD)	32.7 (4.6)	35.3 (1.5)	31.8 (4.3)	31.0 (3.6)	33.2 (3.2)	33.0 (4.4)
	Median	34.0	35.0	32.0	30.0	35.0	31.0
	Min–Max	23‐38	34‐37	25‐39	28‐35	28‐37	30‐38
Body weight, kg	Mean (SD)	66.0 (7.2)	73.4 (2.3)	64.1 (2.4)	63.8 (4.9)	67.0 (8.7)	64.7 (11.9)
	Median	64.2	72.7	63.7	63.8	70.3	61.2
	Min‐max	56.4‐77.8	71.5‐76.0	60.8‐69.0	59.0‐68.7	56.1‐78.4	55.0‐78.0
Height, cm	Mean (SD)	172.5 (7.5)	177.0 (3.6)	171.8 (2.9)	168.9 (3.7)	174.8 (3.6)	170.6 (8.6)
	Median	172.9	179.1	171.0	170.6	174.8	168.0
	Min‐max	158.9‐183.0	172.9‐179.1	168.8‐177.6	164.6‐171.4	170.2‐179.2	163.6‐180.2
BMI, kg/m^2^	Mean (SD)	22.1 (1.0)	23.4 (0.6)	21.7 (1.2)	22.4 (2.0)	21.9 (2.1)	22.1 (1.8)
	Median	22.2	23.7	21.4	23.5	22.7	21.7
	Min‐max	21.1‐24.1	22.7‐23.9	20.2‐24.2	20.1‐23.6	19.0‐24.9	20.5‐24.0

BMI, body mass index; SD, standard deviation.

### Pharmacokinetic Results

#### Single‐Dose Study

**Figure 2:**
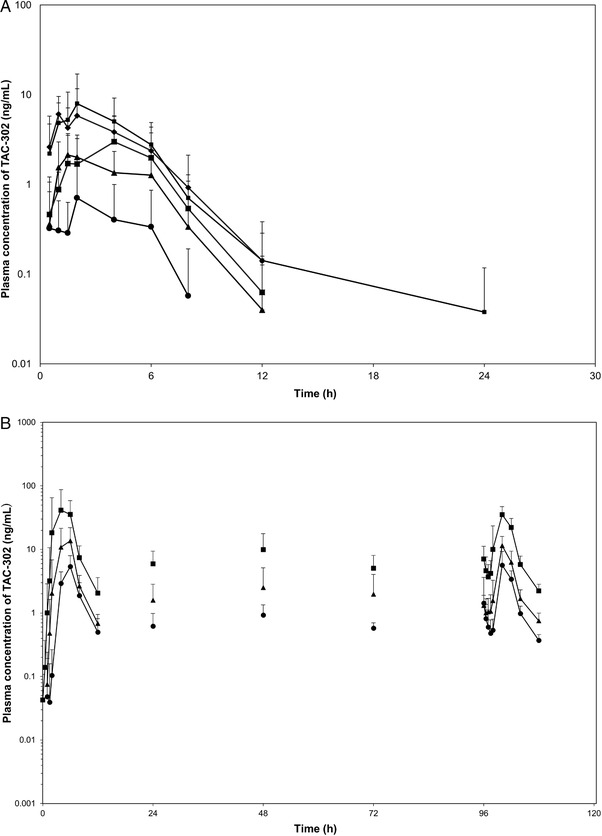
Plasma concentration–time profiles for TAC‐302 after A, a single oral administration of TAC‐302 (single‐dose study; logarithmic plot); and B, multiple‐dose administration (days 7‐11; multiple‐dose study; logarithmic plot). A) •: 100 mg; ▲: 200 mg; ■: 400 mg; ◆: 800 mg; ■: 1200 mg. Mean ± SD (N = 9) B) •: 100 mg; ▲: 200 mg; ■: 400 mg. Mean ± SD (N = 9). SD, standard deviation.

PK parameters for TAC‐302 at each step are listed in Table 3 and shown in Figure 2A. The mean C_max_ after the administration of TAC‐302 at doses of 100, 200, 400, 800, and 1200 mg under fasting conditions were 1.2, 3.2, 3.8, 8.9, and 10.3 ng/mL, respectively. The mean AUC_0‐48h_ and AUC_0‐inf_ at each dose were 3.0 and not calculated, 11.1 and 11.9, 15.7 and 23.9, 30.1 and 29.6, and 35.7 and 35.3 ng • h/mL, respectively. The urinary excretion rate of TAC‐302 and renal clearance were 0.00% and 0 L/h, respectively, and TAC‐302 was not excreted in urine. The C_max_ and AUC_0‐48h_ determined by the linear model satisfied the criteria for linearity. In the fasting state (steps 1, 2, 3A, 4, and 5), dose dependency was evident for C_max_ and AUC. Geometric mean ratios for C_max_ and AUC_0–48h_ after administration of TAC‐302 at a dose of 400 mg in the fed versus fasting states were 12.3 and 13.0, respectively (Table 4). The t_max_ (mean ± SD) value after single oral administration of TAC‐302 400 mg in the fasting versus fed state was 3.6 (1.9) versus 5.3 (1.0) hours.

**Table 3:** PK Parameters for TAC‐302 (Single‐Dose Study)

Step		C_max_ (ng/mL)	t_max_ (h)	AUC_0‐24h_ (ng • h/mL)	AUC_0‐48h_ (ng • h/mL)	AUC_0‐inf_ (ng • h/mL)	t_1/2_ (h)	CL/F (L/h)	A_e_ (%)	CL_r_ (L/h)
1	Mean (SD)	1.2 (1.0)	1.5 (0.6)	3.0 (3.7)	3.0 (3.7)	NC	NC	NC	0 (0.0)	0 (0.0)
	N	9	8	9	9	0	0	0	9	9
2	Mean (SD)	3.2 (1.3)	2.6 (1.7)	11.1 (5.2)	11.1 (5.2)	11.9 (3.3)	1.9 (0.2)	17 900 (5800)	0 (0.0)	0 (0.0)
	N	9	9	9	9	3	3	3	9	9
3A	Mean (SD)	3.8 (2.6)	3.6 (1.9)	15.7 (10.7)	15.7 (10.7)	23.9 (7.6)	1.4 (0.1)	18 100 (6200)	0 (0.0)	0 (0.0)
	N	9	9	9	9	3	3	3	9	9
3B	Mean (SD)	47.1 (34.4)	5.3 (1.0)	173 (85)	178 (88)	158 (72)	4.3 (2.1)	3050 (1460)	0 (0.0)	0 (0.0)
	N	9	9	9	9	8	8	8	9	9
4	Mean (SD)	8.9 (4.7)	1.2 (0.5)	30.1 (13.4)	30.1 (13.4)	29.6 (12.9)	1.5 (0.3)	34 000 (20 300)	0 (0.0)	0 (0.0)
	N	9	9	9	9	9	9	9	9	9
5	Mean (SD)	10.3 (8.3)	1.8 (1.3)	35.2 (26.0)	35.7 (26.4)	35.3 (24.1)	1.6 (0.3)	79 300 (105 000)	0 (0.0)	0 (0.0)
	N	9	9	9	9	5	5	5	9	9

A_e_, urinary excretion rate; AUC_0‐24h_, area under the plasma concentration–time curve from time 0 to 24 hours; AUC_0‐48h_, area under the plasma concentration–time curve from time 0 to 48 hours; AUC_0–inf_, area under the plasma concentration–time curve from time 0 to infinity; CL/F, apparent total clearance of the drug from plasma after oral administration; CL_r_, renal clearance; C_max_, maximum plasma concentration; NC, not calculated; SD, standard deviation; t_max_, time to C_max_; t_1/2_, elimination half‐life.

Fasting: Step 1, 100 mg; Step 2, 200 mg; Step 3A, 400 mg; Step 4, 800 mg; Step 5, 1200 mg.

Fed: Step 3B, 400 mg.

**Table 4:** Effects of an Ordinary Meal on the Pharmacokinetics of TAC‐302 400 mg (Single‐Dose Study)

PK Parameters	Ratio of Geometric Mean Values (Fed Condition/Fasting Condition)	90% Confidence Interval: Lower Limit to Upper Limit
C_max_	12.3	5.67‐26.71
t_max_ a	0.5	0.17‐0.83
AUC_0‐48h_	13.0	6.20‐27.16
AUC_0‐inf_	4.7	1.46‐14.93
t_1/2_	2.9	1.92‐4.40

AUC_0–48h_, area under the plasma concentration–time curve from time 0 to 48 hours; AUC_0–inf_, area under the plasma concentration–time curve from time 0 to infinity; C_max_, maximum plasma concentration; t_max_, time to C_max_; t_1/2_, elimination half‐life.

a The t_max_ is the ratio of the difference in the mean values ([fed] – [fasting]) to the value under the fasting condition.

#### Multiple‐Dose Study

Plasma drug concentration changes for multiple‐dose administration (5 days) of TAC‐302 100 mg, 200 mg, and 400 mg are shown in Figure 2B and Table 5. Correlations were not statistically significant (*P* ≥ .05) between TAC‐302 dose (100–400 mg) and C_max_ or AUC_0–12h_ values in the fitness test result (deviation from the straight line; lack of fit) when assuming the following formula: *Y* = *aX* + *b*. Further, after calculation of point estimates for the intercept (*b*), 95% confidence intervals of intercepts for C_max_ and AUC_0–12h_ included 0 (Table 6). TAC‐302 showed PK linearity up to doses of 400 mg in the fed state. No accumulation of TAC‐302 was observed after repeated‐dose oral administration of TAC‐302 at all doses twice daily for 5 days after the intake of ordinary meals.

**Table 5:** PK Parameters for TAC‐302 After Multiple‐Dose Administration (Multiple‐Dose Study; Repeated‐Dose Period)

	100 mg				200 mg				400 mg			
	C_max_ (ng/mL)	t_max_ (h)	AUC_0‐12h_ (ng•h/mL)	t_1/2_ (h)	C_max_ (ng/mL)	t_max_ (h)	AUC_0‐12h_ (ng•h/mL)	t_1/2_ (h)	C_max_ (ng/mL)	t_max_ (h)	AUC_0‐12h_ (ng•h/mL)	t_1/2_ (h)
Day 7,^a^ mean (SD)	5.9 (1.9)	5.8 (1.2)	23.4 (5.5)	2.4 (NC)	19.6 (6.8)	5.1 (1.1)	61.2 (18.3)	1.5 (0.1)	70.1 (47.2)	4.4 (1.3)	206 (104)	1.5 (0.1)
N	9	9	9	2	9	9	9	3	9	9	9	4
Day 11,^b^ mean (SD)	6.1 (3.1)	4.4 (0.9)	23.8 (8.9)	1.9 (0.2)	11.9 (4.1)	4.2 (0.7)	45.9 (13.5)	2.8 (1.3)	36.6 (10.9)	4.4 (0.9)	157 (36)	2.1 (0.6)
N	9	9	9	4	9	9	9	3	9	9	9	4

AUC_0–12h_, area under the plasma concentration–time curve from time 0 to 12 hours; C_max_, maximum plasma concentration; NC, not calculated; SD, standard deviation; t_max_, time to C_max_; t_1/2_, elimination half‐life.

a Day 7 is the 1st day of multiple‐dose administration.

b Day 11 is the 5th day of multiple‐dose administration.

**Table 6:** Results From the Linear Pharmacokinetic Model of TAC‐302

				(Intercept) b			(Slope) a		
PK Parameter	N	R^2^	LOF *P* Value	Estimated Value	95%CI	*P* Value	Estimated Value	95%CI	*P* Value
Single‐dose study									
C_max_	45	0.39	0.7102	0.95	−1.24 to 3.14	0.3847	0.01	0.01‐0.01	<.0001
AUC_0–48h_	45	0.42	0.6908	3.48	−3.55 to 10.52	0.3236	0.03	0.02‐0.04	<.0001
AUC_0–inf_	20	0.20	0.7874	11.94	−4.82 to 28.69	0.1517	0.02	0.00‐0.04	.0459
Multiple‐dose study									
C_max_	27	0.52	0.5081	−19.30	−42.21 to 3.61	0.0951	0.22	0.13‐0.31	<.0001
AUC_0‐12h_	27	0.64	0.3728	−48.74	−99.72 to 2.23	0.0601	0.62	0.43‐0.82	<.0001

AUC_0–48h_, area under the plasma concentration–time curve from time 0 to 48 hours; AUC_0–inf_, area under the plasma concentration–time curve from time 0 to infinity; AUC_0–12h_, area under the plasma concentration–time curve from time 0 to 12 hours; CI, confidence interval; C_max_, maximum plasma concentration; LOF, lack‐of‐fit; PK, pharmacokinetic.

*Y* = *aX* + *b* (*X*, dose; *Y*, PK parameter).

**Figure 3:**
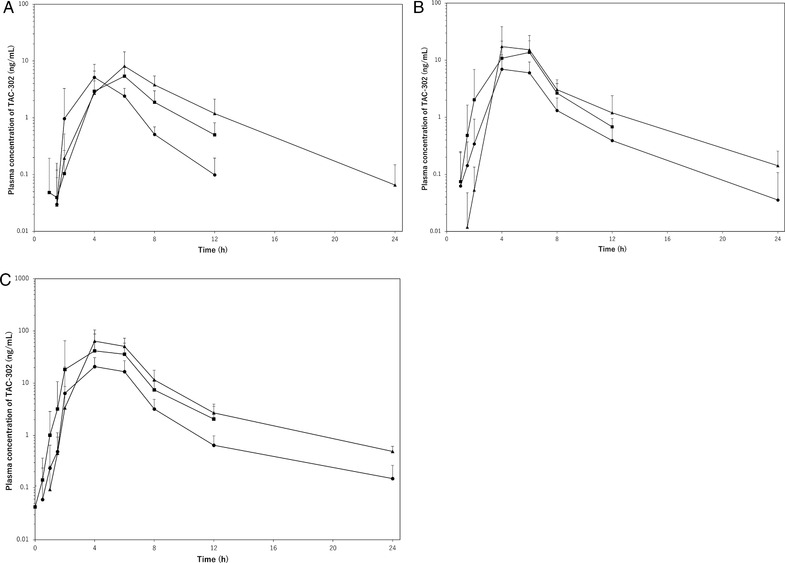
TAC‐302 plasma concentration changes after a low‐fat, high‐fat, or ordinary meal (multiple‐dose study; logarithmic plot) at doses of A, 100 mg; B, 200 mg; and C, 400 mg. **•**: low fat (day 1); ▲: high fat (day 4); ■: ordinary (day 7).

PK parameters for TAC‐302 administration after the intake of low‐fat and high‐fat meals during single‐dose periods 1 and 2, and ordinary meals during the repeated‐dose period are shown in Figure 3A–C and Table 7. Comparing the effect of low‐fat versus high‐fat meals, the C_max_ value showed significant differences after administration of TAC‐302 at doses of 200 mg and 400 mg. That is, with TAC‐302 200 mg, C_max_ increased approximately 3.0‐fold, from 8.9 ng/mL (low‐fat meal) to 26.1 ng/mL (high‐fat meal); the corresponding increase for TAC‐302 400 mg was approximately 3.5‐fold, from 24.6 to 80.3 ng/mL. Thus, the plasma concentration of TAC‐302 was judged to increase in association with an increased fat content of the meal. The AUC_0–12h_ value showed significant differences at all doses. With TAC‐302 100 mg, the AUC_0–12h_ approximately doubled, from 18.1 ng • h/mL (low‐fat meal) to 35.7 ng • h/mL (high‐fat meal); the corresponding increase for TAC‐302 200 mg was approximately 2.5‐fold, from 31.2 to 76.6 ng • h/mL; and the increase for TAC‐302 400 mg was approximately 3.0 fold, from 93.8 to 274 ng • h/mL (Table 7). Thus, the AUC_0–12h_ value for TAC‐302 was considered to increase in association with an increased fat content of the meal.

**Table 7:** Comparison of PK Parameters for TAC‐302 Administration After a Low‐Fat, High‐Fat, or Ordinary Meal

	100 mg				200 mg				400 mg			
	C_max_ (ng/mL)	t_max_ (h)	AUC_0‐12h_ (ng • h/mL)	t_1/2_ (h)	C_max_ (ng/mL)	t_max_ (h)	AUC_0‐12h_ (ng • h/mL)	t_1/2_ (h)	C_max_ (ng/mL)	t_max_ (h)	AUC_0‐12h_ (ng • h/mL)	t_1/2_ (h)
Low‐fat meal (day 1) Mean (SD) N	5.9 (3.2) 9	4.2 (1.2) 9	18.1 (7.6) 9	1.5 (0.2) 3	8.9 (4.3) 9	4.7 (1.0) 9	31.2 (15.4) 9	2.3 (1.5) 7	24.6 (12.4) 9	4.0 (1.0) 9	93.8 (41.6) 9	2.9 (1.6) 5
High‐fat meal (day 4) Mean (SD) N	9.1 (5.3) 9	7.1 (2.0) 9	35.7 (17.8) 9	3.8 (NC) 2	26.1 (18.6) 9	5.1 (1.1) 9	76.6 (47.2) 9	4.4 (0.8) 4	80.3 (25.5) 9	4.7 (1.0) 9	274 (71) 9	3.9 (0.7) 7
Ordinary meal (day 7) Mean (SD) N	5.9 (1.9) 9	5.8 (1.2) 9	23.4 (5.5) 9	2.4 (NC) 2	19.6 (6.8) 9	5.1 (1.1) 9	61.2 (18.3) 9	1.5 (0.1) 3	70.1 (47.2) 9	4.4 (1.3) 9	206 (104) 9	1.5 (0.1) 4

AUC_0–12h_, area under the plasma concentration–time curve from time 0 to 12 hours; C_max_, maximum plasma concentration; NC, not calculated; t_max_, time to C_max_; SD, standard deviation; t_1/2_, elimination half‐life.

### Safety

No deaths or serious or severe AEs occurred in either study, and none of the participants discontinued the study because of AEs. Regarding 12‐lead electrocardiogram, blood pressure, pulse rate, and body temperature, there were no clinically relevant changes in the TAC‐302 groups.

#### Single‐Dose Study

In the single‐dose study, the numbers of participants with AEs after TAC‐302 administration in the fasting state were 2 of 9 at 100 mg, 4 of 9 at 200 mg, 4 of 9 at 400 mg, 2 of 9 at 800 mg, and 2 of 9 at 1200 mg. In the placebo group, 7 of 15 participants had AEs. In the fed state, AEs occurred in 5 of 9 participants in the TAC‐302 400‐mg group, and in 1 of 3 participants in the placebo group. In the TAC‐302 group, moderate urethritis occurred in 1 of 9 participants after administration at 1200 mg under the fasting condition. The investigator regarded the event as an accidental infection and evaluated it as having no reasonable causal relationship with TAC‐302. All AEs observed except for this urethritis were mild and resolved without treatment. There were no major noteworthy ADRs after TAC‐302 administration in both the fasting and fed states, and all AEs that were regarded as ADRs in the TAC‐302 or placebo group were mild in severity and resolved without treatment.

##### Multiple‐Dose Study

AEs reported in the multiple‐dose study according to severity are shown in Table S1. The numbers of participants who experienced AEs in the TAC‐302 group during the repeated‐dose period were 4 of 9 at 100 mg, 2 of 9 at 200 mg, and 4 of 9 at 400 mg. In the placebo group, 4 of 9 participants had AEs. In the multiple‐dose study, 2 of 9 participants in the TAC‐302 400‐mg dose group had an ADR (*N*‐acetyl‐*β*‐D‐glucosaminidase increased and free tri‐iodothyronine increased, 1 participant each).

The numbers of participants who experienced AEs in the TAC‐302 group during single‐dose period 1 (low‐fat meals) were 6 of 9 participants at 100 mg, 1 of 9 participants at 200 mg, and 6 of 9 participants at 400 mg. All AEs observed in single‐dose period 1 were mild and resolved without treatment.

The numbers of participants who experienced AEs in the TAC‐302 group during single‐dose period 2 (high‐fat meals) were 2 of 9 participants at 100 mg, 1 of 9 participants at 200 mg, and 2 of 9 participants at 400 mg.

## Discussion

Herein, we report the results of the 2 first‐in‐human phase I studies of TAC‐302, which showed the novel pharmacological action of TAC‐302 with an acceptable safety profile in healthy male participants.

Our study showed linear PK for TAC‐302 after administration of 100 to 1200 mg in a fasting state in the single‐dose study. In the multiple‐dose study, linear PK was also identified with the administration of TAC‐302 100–400 mg after participants had consumed an ordinary meal. That is, C_max_ and AUC_0–12h_ satisfied the criteria for linearity in the linear model. No accumulation of TAC‐302 was noted with multiple‐dose administration at any dose. No difference was observed in the incidence of AEs between the TAC‐302 group and the placebo group in both single‐dose and repeated‐dose studies. TAC‐302 was not associated with drug accumulation, and no new safety concerns were raised.

Exposure to TAC‐302 increased when a single oral dose was administered after meals. Indeed, geometric mean ratios for C_max_ and AUC_0–48h_ in the fed versus fasting state were 12.3 and 13.0, respectively. Thus, we investigated meal fat content in the multiple‐dose study. Significant differences were evident in C_max_ after TAC‐302 200 mg and 400 mg and in the AUC_0–12h_ at all doses, and mean values after a high‐fat meal were approximately 2 to 3 times greater than those after a low‐fat meal. In the literature, few reports exist about the influence of meal fat content on drug PK. Nevertheless, one study in healthy Japanese male volunteers revealed that the oral bioavailability of the highly lipophilic drug menatetrenone (vitamin K_2_) increased in line with increased meal fat content: that is, AUC_0–24h_ increased by about 1.5‐fold with an approximate doubling of meal fat content and increased by about 3.0‐fold with an approximate quadrupling of meal fat content. Importantly, the increase in bioavailability appeared to peak when meal fat content was >35 g.^10^ Previous studies in non‐Japanese healthy volunteers also demonstrated that increased meal fat content increased the oral bioavailability of dalcetrapib and fenretinide.^11^, ^12^ Although plasma TAC‐302 concentration increases with increasing meal fat content, these increases were no different than those reported previously.^10^

Despite the fact that plasma TAC‐302 concentrations in the fed state were approximately 10 times greater than those in the fasting state, there were no differences in the incidences of AEs between the 2 conditions. Regarding meal fat content, the incidence of AEs after a single administration of TAC‐302 was higher after a low‐fat meal than after a high‐fat meal (48.1% and 18.5%, respectively). Therefore, no correlation was evident between plasma TAC‐302 concentrations and the incidence of AEs, and differences in plasma TAC‐302 concentrations were not considered to affect the overall safety profile of TAC‐302.

In the multiple‐dose study, regardless of the type of meal the AUC_0‐12h_ of TAC‐302 200 mg administered twice daily after meals reached an equivalent amount of exposure to 10 mg/kg (bid) administered orally in a nonclinical study,^13^ where maximum efficacy was confirmed in a rat diabetic dysuria model. Based on this, we considered that repeated administration of TAC‐302 200 mg twice daily in the morning and after dinner was expected to be clinically effective.

These studies have confirmed that TAC‐302 has good tolerability and safety, and acceptable PK, so we are conducting a phase 2 study to determine the efficacy and safety of TAC‐302 in patients with overactive bladder who have detrusor underactivity (Clinicaltrials.gov identifier: NCT03175029).

The principle limitation of the current studies is that, because we evaluated the PK of TAC‐302 in healthy Japanese men, the results cannot be extrapolated to other ethnic groups. Additional limitations include the small sample size and single‐center design; more detailed safety evaluations will be conducted in future, larger‐scale clinical studies.

## Conclusions

TAC‐302 showed acceptable tolerability, safety, and PK linearity up to doses of 1200 mg in the fasting state and across the dose range of 100 to 400 mg in the fed state, and no accumulation was observed. Although TAC‐302 PK was influenced by meal fat content, TAC‐302 had a wide safety margin. Given its anticipated favorable safety profile and unique mechanism of action, TAC‐302 may develop into a novel therapeutic agent for the management of patients with LUTD.

## Conflicts of Interest

J.H., T.O., E.N., and K.M. are employees of Taiho Pharmaceutical and own Otsuka Holdings stock. S.S. declares no conflict of interest.

## Funding

These studies were supported by Taiho Pharmaceutical Co., Ltd., Tokyo, Japan.

## Author Contributions

S.S., the principal study investigator, contributed to the conduct of the study and data collection. J.H., T.O., E.N., and K.M. contributed to the study concept and design, conduct of the study, data analysis, and interpretation. All authors contributed to manuscript preparation, manuscript review and revisions, and final approval of the manuscript.

## Supporting information

Supplemental Information

Supplemental Information
