# Supplementary material for: Safety, Tolerability, Pharmacokinetics, and Food Effects on TAC‐302 in Healthy Participants: Randomized, Double‐Blind, Placebo‐Controlled, Single‐Dose and Multiple‐Dose Studies
Source: Clin Pharmacol Drug Dev. 2020 Jan 22;9(7):821–32. doi: 10.1002/cpdd.776 (PMC7586813; doi:10.1002/cpdd.776)
Supplement: Supplementary file 1 — Supplemental Information [file CPDD-9-821-s002.docx]

**Supplemental On-Line Content**

**Table S1.** Incidence of AEs according to severity (multiple-dose study)

| Dose | MedDRA (ver.16.1) System Organ Class | | TAC-302 group | | | | Placebo group | | | |
| --- | --- | --- | --- | --- | --- | --- | --- | --- | --- | --- |
|  |  |  | Total  n (%) | Severity | | | Total  n (%) | Severity | | |
|  |  |  |  | Mild  n (%) | Moderate  n (%) | Severe  n (%) |  | Mild  n (%) | Moderate  n (%) | Severe  n (%) |
|  |  | Preferred Term |  |  |  |  |  |  |  |  |
| 100 mg |  | | (N=9) |  | | | (N=3) |  | | |
|  | Adverse events of any kind | | 4 (44.4) | 4 (44.4) | 0 (0.0) | 0 (0.0) | 1 (33.3) | 1 (33.3) | 0 (0.0) | 0 (0.0) |
|  | Infections and infestations | | 1 (11.1) | 1 (11.1) | 0 (0.0) | 0 (0.0) | 0 (0.0) | 0 (0.0) | 0 (0.0) | 0 (0.0) |
|  |  | Pharyngitis | 1 (11.1) | 1 (11.1) | 0 (0.0) | 0 (0.0) | 0 (0.0) | 0 (0.0) | 0 (0.0) | 0 (0.0) |
|  | Investigations | | 3 (33.3) | 3 (33.3) | 0 (0.0) | 0 (0.0) | 1 (33.3) | 1 (33.3) | 0 (0.0) | 0 (0.0) |
|  |  | Amylase increased | 0 (0.0) | 0 (0.0) | 0 (0.0) | 0 (0.0) | 1 (33.3) | 1 (33.3) | 0 (0.0) | 0 (0.0) |
|  |  | Blood glucose increased | 1 (11.1) | 1 (11.1) | 0 (0.0) | 0 (0.0) | 0 (0.0) | 0 (0.0) | 0 (0.0) | 0 (0.0) |
|  |  | Blood triglycerides increased | 1 (11.1) | 1 (11.1) | 0 (0.0) | 0 (0.0) | 0 (0.0) | 0 (0.0) | 0 (0.0) | 0 (0.0) |
|  |  | Free tri-iodothyronine increased | 1 (11.1) | 1 (11.1) | 0 (0.0) | 0 (0.0) | 0 (0.0) | 0 (0.0) | 0 (0.0) | 0 (0.0) |
| 200 mg |  | | (N=9) |  | | | (N=3) |  | | |
|  | Adverse events of any kind | | 2 (22.2) | 2 (22.2) | 0 (0.0) | 0 (0.0) | 1 (33.3) | 1 (33.3) | 0 (0.0) | 0 (0.0) |
|  | Investigations | | 2 (22.2) | 2 (22.2) | 0 (0.0) | 0 (0.0) | 1 (33.3) | 1 (33.3) | 0 (0.0) | 0 (0.0) |
|  |  | *N*-acetyl-*β*-D-glucosaminidase increased | 0 (0.0) | 0 (0.0) | 0 (0.0) | 0 (0.0) | 1 (33.3) | 1 (33.3) | 0 (0.0) | 0 (0.0) |
|  |  | Blood creatine phosphokinase increased | 1 (11.1) | 1 (11.1) | 0 (0.0) | 0 (0.0) | 0 (0.0) | 0 (0.0) | 0 (0.0) | 0 (0.0) |
|  |  | Blood lactate dehydrogenase increased | 1 (11.1) | 1 (11.1) | 0 (0.0) | 0 (0.0) | 0 (0.0) | 0 (0.0) | 0 (0.0) | 0 (0.0) |
|  |  | C-reactive protein increased | 1 (11.1) | 1 (11.1) | 0 (0.0) | 0 (0.0) | 0 (0.0) | 0 (0.0) | 0 (0.0) | 0 (0.0) |
| 400 mg |  | | (N=9) |  | | | (N=3) |  | | |
|  | Adverse events of any kind | | 4 (44.4) | 4 (44.4) | 0 (0.0) | 0 (0.0) | 2 (66.7) | 2 (66.7) | 0 (0.0) | 0 (0.0) |
|  | Investigations | | 4 (44.4) | 4 (44.4) | 0 (0.0) | 0 (0.0) | 2 (66.7) | 2 (66.7) | 0 (0.0) | 0 (0.0) |
|  |  | *N*-acetyl-*β*-D-glucosaminidase increased | 1 (11.1) | 1 (11.1) | 0 (0.0) | 0 (0.0) | 0 (0.0) | 0 (0.0) | 0 (0.0) | 0 (0.0) |
|  |  | Blood thyroid stimulating hormone increased | 1 (11.1) | 1 (11.1) | 0 (0.0) | 0 (0.0) | 1 (33.3) | 1 (33.3) | 0 (0.0) | 0 (0.0) |
|  |  | C-reactive protein increased | 1 (11.1) | 1 (11.1) | 0 (0.0) | 0 (0.0) | 0 (0.0) | 0 (0.0) | 0 (0.0) | 0 (0.0) |
|  |  | Glucose urine present | 0 (0.0) | 0 (0.0) | 0 (0.0) | 0 (0.0) | 1 (33.3) | 1 (33.3) | 0 (0.0) | 0 (0.0) |
|  |  | Free tri-iodothyronine increased | 1 (11.1) | 1 (11.1) | 0 (0.0) | 0 (0.0) | 1 (33.3) | 1 (33.3) | 0 (0.0) | 0 (0.0) |
|  |  | Thyroxine free decreased | 1 (11.1) | 1 (11.1) | 0 (0.0) | 0 (0.0) | 0 (0.0) | 0 (0.0) | 0 (0.0) | 0 (0.0) |
|  | Reproductive system and breast disorders | | 0 (0.0) | 0 (0.0) | 0 (0.0) | 0 (0.0) | 1 (33.3) | 1 (33.3) | 0 (0.0) | 0 (0.0) |
|  |  | Genital discomfort | 0 (0.0) | 0 (0.0) | 0 (0.0) | 0 (0.0) | 1 (33.3) | 1 (33.3) | 0 (0.0) | 0 (0.0) |
| All doses |  | | (N=27) |  | | | (N=9) |  | | |
|  | Adverse events of any kind | | 10 (37.0) | 10 (37.0) | 0 (0.0) | 0 (0.0) | 4 (44.4) | 4 (44.4) | 0 (0.0) | 0 (0.0) |
|  | Infections and infestations | | 1 (3.7) | 1 (3.7) | 0 (0.0) | 0 (0.0) | 0 (0.0) | 0 (0.0) | 0 (0.0) | 0 (0.0) |
|  |  | Pharyngitis | 1 (3.7) | 1 (3.7) | 0 (0.0) | 0 (0.0) | 0 (0.0) | 0 (0.0) | 0 (0.0) | 0 (0.0) |
|  | Investigations | | 9 (33.3) | 9 (33.3) | 0 (0.0) | 0 (0.0) | 4 (44.4) | 4 (44.4) | 0 (0.0) | 0 (0.0) |
|  |  | Amylase increased | 0 (0.0) | 0 (0.0) | 0 (0.0) | 0 (0.0) | 1 (11.1) | 1 (11.1) | 0 (0.0) | 0 (0.0) |
|  |  | *N*-acetyl-*β*-D-glucosaminidase increased | 1 (3.7) | 1 (3.7) | 0 (0.0) | 0 (0.0) | 1 (11.1) | 1 (11.1) | 0 (0.0) | 0 (0.0) |
|  |  | Blood creatine phosphokinase increased | 1 (3.7) | 1 (3.7) | 0 (0.0) | 0 (0.0) | 0 (0.0) | 0 (0.0) | 0 (0.0) | 0 (0.0) |
|  |  | Blood glucose increased | 1 (3.7) | 1 (3.7) | 0 (0.0) | 0 (0.0) | 0 (0.0) | 0 (0.0) | 0 (0.0) | 0 (0.0) |
|  |  | Blood lactate dehydrogenase increased | 1 (3.7) | 1 (3.7) | 0 (0.0) | 0 (0.0) | 0 (0.0) | 0 (0.0) | 0 (0.0) | 0 (0.0) |
|  |  | Blood thyroid stimulating hormone increased | 1 (3.7) | 1 (3.7) | 0 (0.0) | 0 (0.0) | 1 (11.1) | 1 (11.1) | 0 (0.0) | 0 (0.0) |
|  |  | Blood triglycerides increased | 1 (3.7) | 1 (3.7) | 0 (0.0) | 0 (0.0) | 0 (0.0) | 0 (0.0) | 0 (0.0) | 0 (0.0) |
|  |  | C-reactive protein increased | 2 (7.4) | 2 (7.4) | 0 (0.0) | 0 (0.0) | 0 (0.0) | 0 (0.0) | 0 (0.0) | 0 (0.0) |
|  |  | Glucose urine present | 0 (0.0) | 0 (0.0) | 0 (0.0) | 0 (0.0) | 1 (11.1) | 1 (11.1) | 0 (0.0) | 0 (0.0) |
|  |  | Free tri-iodothyronine increased | 2 (7.4) | 2 (7.4) | 0 (0.0) | 0 (0.0) | 1 (11.1) | 1 (11.1) | 0 (0.0) | 0 (0.0) |
|  |  | Thyroxine free decreased | 1 (3.7) | 1 (3.7) | 0 (0.0) | 0 (0.0) | 0 (0.0) | 0 (0.0) | 0 (0.0) | 0 (0.0) |
|  | Reproductive system and breast disorders | | 0 (0.0) | 0 (0.0) | 0 (0.0) | 0 (0.0) | 1 (11.1) | 1 (11.1) | 0 (0.0) | 0 (0.0) |
|  |  | Genital discomfort | 0 (0.0) | 0 (0.0) | 0 (0.0) | 0 (0.0) | 1 (11.1) | 1 (11.1) | 0 (0.0) | 0 (0.0) |

MedDRA, Medical Dictionary for Regulatory Activities
