# Supplementary material for: Safety, Tolerability, Pharmacokinetics, and Food Effects on TAC‐302 in Healthy Participants: Randomized, Double‐Blind, Placebo‐Controlled, Single‐Dose and Multiple‐Dose Studies
Source: Clin Pharmacol Drug Dev. 2020 Jan 22;9(7):821–32. doi: 10.1002/cpdd.776 (PMC7586813; doi:10.1002/cpdd.776)
Supplement: Supplementary file 2 — Supplemental Information [file CPDD-9-821-s001.pptx]

## Slide 1
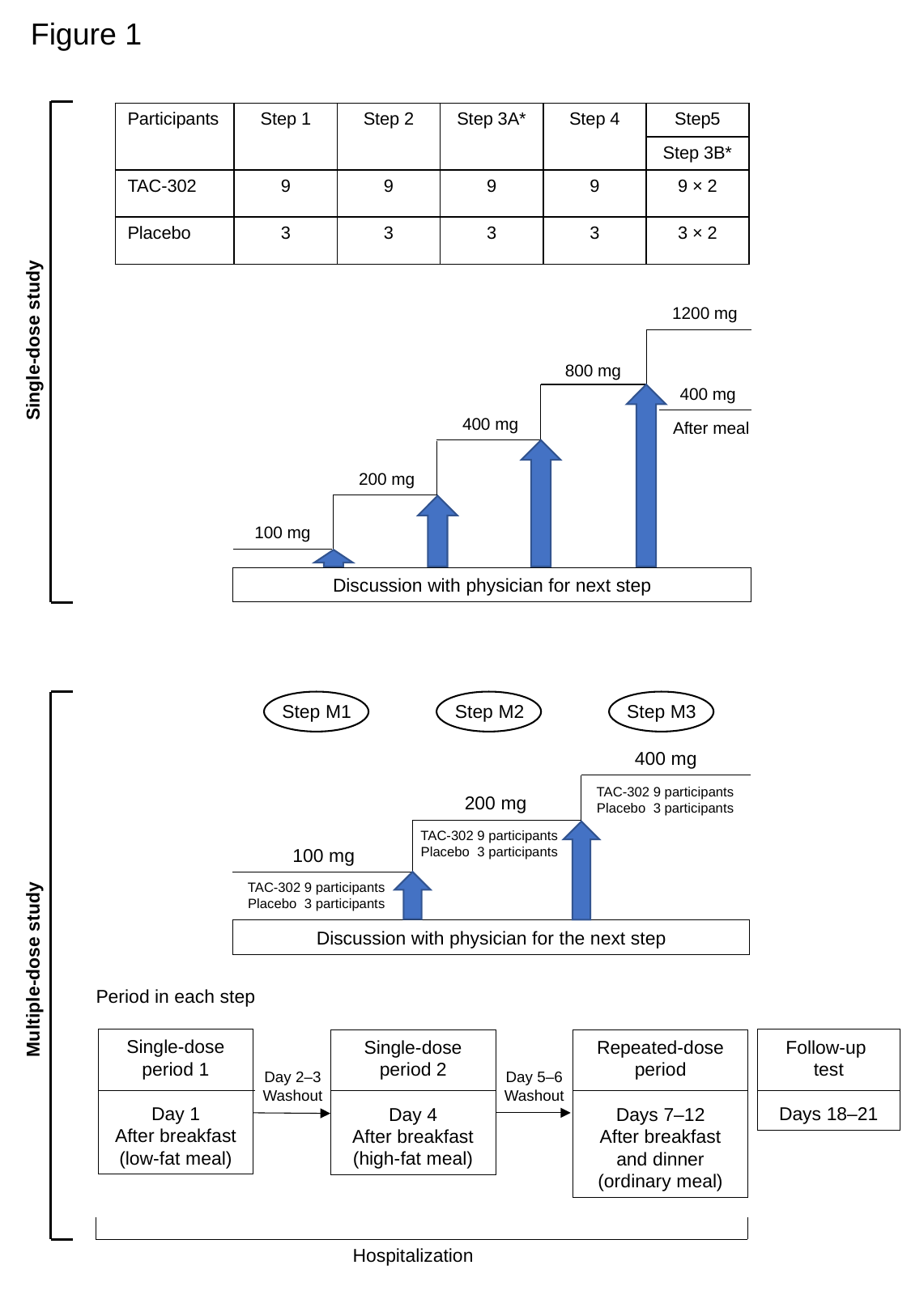

Figure 1
| Participants | Step 1 | Step 2 | Step 3A\* | Step 4 | Step5 |
| --- | --- | --- | --- | --- | --- |
| | | | | | Step 3B\* |
| TAC-302 | 9 | 9 | 9 | 9 | 9 × 2 |
| Placebo | 3 | 3 | 3 | 3 | 3 × 2 |
1200 mg
Single-dose study
800 mg
400 mg
400 mg
After meal
200 mg
100 mg
Discussion with physician for next step
Step M1
Step M2
Step M3
400 mg
TAC-302 9 participants
Placebo 3 participants
200 mg
TAC-302 9 participants
Placebo 3 participants
100 mg
TAC-302 9 participants
Placebo 3 participants
Discussion with physician for the next step
Multiple-dose study
Period in each step
Single-dose period 1
Day 1
After breakfast
(low-fat meal)
Follow-up
test
Days 18–21
Repeated-dose period
Days 7–12
After breakfast and dinner
(ordinary meal)
Single-dose period 2
Day 4
After breakfast
(high-fat meal)
Day 2–3
Washout
Day 5–6
Washout
Hospitalization
